# Iron and Ferritin Levels in Saliva of Patients with Thalassemia and Iron Deficiency Anemia

**DOI:** 10.4084/MJHID.2012.051

**Published:** 2012-08-09

**Authors:** Duran Canatan, Sevgi Kosaci Akdeniz

**Affiliations:** Suleyman Demirel University, Department of Pediatric Hematology, Isparta, Turkey

## Abstract

Most of the techniques for measuring iron stores such as serum iron concentration, iron binding capacity, serum ferritin level, liver biopsy can be troublesome or invasive for patients with thalassemia. The salivary iron measurement could be of potential advantage being an easy and non invasive approach for diagnosis of iron deficiency and iron overload . The aim of this study was to compare the levels of iron and ferritin in saliva and serum of patients affected by thalassemia or iron deficiency anemia. For this purpose, 96 patients with iron overload (71 with thalassemia major, 10 with thalassemia intermedia and 15 with thalassemia trait), 30 patients with iron deficiency anemia, and 35 healthy children as control group were involved in this study. Their saliva and serum iron and ferritin levels were measured. Iron and ferritin levels were higher in iron overload groups than in control group and lower in iron deficiency group (p<0.05). Furthermore serum and saliva iron and ferritin levels paralleled in all groups. In conclusion, iron and ferritin saliva can be routinely used for diagnosis of both iron overload and deficiency; furthermore this procedure may be an important advantage for blood donors being easily available and not invasive.

## Introduction

An accurate assessment of body iron stores is essential for the diagnosis and therapy of iron overload in thalassemia. Most of the techniques for measuring iron stores such as serum iron concentration, iron binding capacity, serum ferritin level, liver biopsy are troublesome and/or invasive.^1^ Iron deficiency anemia is also the most common anemia in childhood and repeated measurement of iron ad ferritin level are required for its diagnosis and follow-up.^2^,^3^ The salivary iron measurement may be the potential advantage of an easy and non invasive approach in iron deficient and iron overload conditions.^4^–^6^ Pharmacokinetic profiles of some drugs such as L1 is an iron chelator has been shown similar in serum and saliva.^7^ The aim of this study was to compare the levels of iron and ferritin in saliva and serum of patients with thalassemia and iron deficiency anemia.

## Material and methods

This study was carried out at Department of Pediatrics of Suleyman Demirel University. The study was reviewed and approved by the Ethical Committee of Suleyman Demirel

### University Faculty of Medicine

The study was carried out in accordance with the ethical standards laid down in the World Medical Association Declaration of Helsinki.

The study population was selected from the patients with iron overload (thalassemia major, intermedia, trait) and iron deficiency anemia : 71 patients with transfusion dependent β-thalassemia major (37 female, 34 male) aged between 6–23 (11.6±4.45) years; 10 patients with thalassemia intermedia (5 female, 5 male) aged between 6–25 (12.4±6.0) years; 15 patients with thalassemia trait (5 female, 10 male) aged between 15–18 (16.0 ±1.9) years; 30 patients with iron deficiency, 17 female and 13 male, aged between 7–14 (9.1±2.12) years. The control group consisted of sex and age matched subjects. The control group was formed by 34 subjects aged between 7–15 (10±2.22) years. All of the subjects enrolled in the study and their parents were informed about the study procedures and a written consent was signed by all of the participants.

All patients were examined before collecting saliva for periodontal disease, gum and oral bleeding. The patients washed out their mouth with distilled water before starting the procedure. Non-stimulative saliva was collected by a standard spit method as the patient, at sitting position and prone to front, spits for 30 minutes in a plastic container. The saliva was transferred from the plastic container to glass tubes and then centrifuged for 10 minutes at 4000 rpm.^5^ Iron; iron binding capacity and ferritin were tested in saliva and serum samples with the same method in the same day.^2^

The statistical analyses were performed by SPSS 15 (SPSS Inc. IL, USA) package programme. We used Mann-Whitney U test, Chi-Square test, and Pearson and Spearman correlation co-efficient. p<0.05 was accepted as statistically significant.

## Results

A total of 160 subjects, 71 affected by thalassemia major, 10 by thalassemia intermedia, 15 by thalassemia trait, 30 by iron deficiency and 34 healthy peoples were involved. There was no statistically significant difference between study groups and control group for age and gender (p>0.05). Saliva iron and ferritin levels of all study groups were statistically significantly different from control group (p<0.05). Saliva iron binding capacity was only significantly higher in the iron deficiency group (p<0.05). Saliva ferritin levels were highest in thalassemia major and intermedia group and lowest in iron deficiency anemia group (Table 1).

There was a statistically significant positive correlation between serum iron, ferritin and saliva iron, ferritin levels (p<0.05) in all groups (Table 2). Median values of serum and saliva iron, iron binding capacity and ferritin levels of all groups are seen at Table 3. Median saliva/serum iron ratio of study and control group, there was no difference in thalassemia group, but there was 1.5 times in iron deficiency group. Median saliva/serum ferritin ratio of study and control group compared, there was 0.6 and 2 times greater in thalassemia and iron deficiency than control group. Median saliva/serum iron binding capacity ratio of study and control group compared, it was similar in thalassemia and control group and 2 times greater in iron deficiency.

## Discussion

Diagnostic use of saliva is well accepted by physicians and patients being an easy and non-invasive approach. 4–6 Borgna-Pignatti at al.^8^ published a thalassemia major patient with Sicca Syndrome having hemosiderin at parenchymal cells of minor salivary gland biopsy material ; iron overload was attributed to multiple transfusions. Mishra at al.^9^ compared the serum and saliva levels of iron in 40 anemic patients and 10 healthy children. Their study showed a high correlation between serum and saliva iron levels both for iron deficient and iron overloaded groups. We also found a high correlation in thalassemic patients but a moderate correlation between serum and saliva iron levels in iron deficient group.

There was no study about comparison of ferritin levels at saliva and serum in the literature. In our study, we compared ferritin levels at all groups and found a moderate correlation between serum and saliva ferritin levels in thalassemic group.

Iron stores deficiency is a common side effect of whole blood donation. 10 The measurement of ferritin in saliva before donation may permit early recognition while reducing blood loss .

## Conclusion

Saliva iron and ferritin levels increase as well as serum in patients with thalassemia and decrease in patients with iron deficiency anemia. Saliva can be used for diagnosis routinely to show the iron overload and deficiency of the body; furthermore its easy applicability and also a non-invasive procedure may be an important advantage for blood donors and children.

## Figures and Tables

**Table 1:** Serum and saliva iron, serum iron binding capacity, ferritin levels in patients

	Control	Thalassemia Major	Thalassemia intermedia	Thalassemia Trait	Iron Deficiency Anemia
N	34	71	10	15	30
Serum iron (mg/dl)	62.37±33	192.25±76.87 (p<0.05)	132.200±55.7 (p<0.05)	93.4±39.14 (p<0.05)	13.8±24.7 (p<0.05)
Serum iron binding capacity (mg/dl)	335.82±95	333.32±70.53 (p>0.05)	334.1±67.4 (p>0.05)	310.9±95.4 (p>0.05)	366.3±55.7 (p<0.05)
Serum ferritin (ng/dl)	29,67 ± 16,78	2826,35 ±1711 (p<0.05)	1492,1 ± 1445,1 (p<0.05)	126 ± 150 (p<0.05)	6,7 ± 3,49 (p<0.05)
Saliva iron (mg/dl)	74,20 ± 40,7	253,64 ± 91, (p<0.05)	150,1 ± 61,1 (p<0.05)	101,1 ± 41,16 (p<0.05)	24,6 ± 10 (p<0.05)
Saliva iron binding capacity (mg/dl)	382,20 ± 94,77	396,74 ± 92,96 (p>0.05)	434,7 ± 82,6 (p>0.05)	362,6 ± 65,23 (p>0.05)	792,2 ± 151,1 (p<0.05)
Saliva ferritin (ng/dl)	42,5 ± 42,25	2529,6 ± 1081,3 (p<0.05)	1166 ± 951,3 (p<0.05)	112,2 ± 145,9 (p<0.05)	18,6 ± 8,53 (p<0.05)

**Table 2:** Correlation levels between serum and saliva iron and ferritin

	Serum - saliva iron	Serum - saliva Ferritin
Control	r =0.885, p=0.000*	r =0.842, p=0.000*
Thalassemia Major	r =0.972, p=0.000*	r =0.364, p=0.034*
Thalassemia Intermedia	r =0.720, p=0.019**	r =0.891, p=0.001**
Thalassemia Trait	r =0.955, p=0.000**	r =0.831, p=0.000**
Iron Deficiency Anemia	r =0.368, p=0.045*	r =0.880, p=0.000*

* pearson correlation,

** spearman correlation

**Table 3:** Median ratio of saliva and serum iron, ferritin and serum iron binding capacity

	Median saliva /serum iron ratio	Median saliva /serum ferritin ratio	Median saliva /serum iron binding capacity ratio
Control	1,2	1.4	1.1
Thalassemia Major	1,3	0.8	1.1
Thalassemia Intermedia	1.1	0.8	1.2
Thalassemia Trait	1.1	0.8	1.1
Iron Deficiency Anemia	1.8	2.7	2.1
